# Terrestrial land use signals on groundwater fauna beyond current protection buffers

**DOI:** 10.1002/eap.3040

**Published:** 2024-10-18

**Authors:** Mara Knüsel, Roman Alther, Florian Altermatt

**Affiliations:** ^1^ Department of Aquatic Ecology, Eawag Swiss Federal Institute of Aquatic Science and Technology Dübendorf Switzerland; ^2^ Department of Evolutionary Biology and Environmental Studies University of Zurich Zurich Switzerland

**Keywords:** land cover, meta‐ecosystem, *Niphargus*, pollution, stygofauna, subterranean, water quality

## Abstract

Terrestrial and aquatic ecosystems are tightly linked, with direct implications for applied resource management and conservation. It is well known that human land use change and intensification of terrestrial systems can have large impacts on surface freshwater ecosystems. Contrastingly, the study and understanding of such land use impacts on groundwater communities is lagging behind. Both the impact strength of land use on groundwater communities and the spatial extents at which such interlinkages are operating are largely unknown, despite our reliance on groundwater for drinking water extraction as a key ecosystem service. Here, we analyzed groundwater amphipod occurrence from several hundred shallow groundwater aquifers used for drinking water extraction across a region of varying agricultural intensity and human population density in Switzerland. Despite drinking water extraction sites being generally built at locations with expected minimal aboveground impacts on water quality, we found a direct correlation between land use type and intensity within the surrounding catchment area and the locally measured nitrate concentrations, which is a direct proxy for drinking water quality. Furthermore, groundwater amphipods were more likely to be found at sites with higher forest coverage than at sites with higher crop and intensive pasture coverages, clearly indicating a tight connection between aboveground land use and groundwater biodiversity. Our results indicate that land use type effects on groundwater communities are most relevant and pronounced to spatial scales of about 400–1000 m around the groundwater sampling site. Importantly, the here identified spatial scale is 1.2‐ to 3‐fold exceeding the average extent of currently defined groundwater protection zones. We postulate that incorporating an ecosystem perspective into groundwater management strategies is needed for effective protection of groundwater quality and biodiversity.

## INTRODUCTION

Terrestrial and aquatic ecosystems are tightly linked through cross‐ecosystem spatial flows of resources and pollutants alike (Burdon et al., 2019; Gounand et al., 2018; Munz et al., 2017). Such landscape‐level processes can have large impacts on local ecological conditions and communities (Knight et al., 2005). As freshwater ecosystems such as streams and lakes are embedded in a terrestrial matrix, surrounding land use can strongly affect local water quality and biological assemblages through spatial flows of nutrients and contaminants from agricultural and urbanized areas (e.g., Allan, 2004; Cereghetti & Altermatt, 2023; Kirschner et al., 2023).

Yet, when studying cross‐ecosystem effects between terrestrial and aquatic realms, groundwater ecosystems are very commonly overlooked. This is surprising, as groundwater is by far the earth's largest liquid freshwater reserve (Gleeson et al., 2016), and of highest relevancy for humans for drinking water access. Saccò et al. (2024) showed that over half of all land surface areas have substantial interactions with groundwater. Through groundwater discharge, valuable services are provided to groundwater‐dependent ecosystems such as springs, streams, and wetlands (Kløve et al., 2011). Groundwater also plays a vital role in sustaining human life. It serves as a reliable and accessible source of drinking water for a significant portion of the global population (United Nations, 2022). Beyond drinking water, groundwater is essential for supporting agriculture, industrial processes, and overall economic activities (Giordano, 2009). Finally, groundwater ecosystems contain an exceptional and often very enigmatic diversity of organisms (Deharveng et al., 2009). This diversity and the occurrence of microbial and macrobial species is directly linked to the purification of drinking water and respective ecosystem services (Griebler & Avramov, 2015). Although likely less studied than surface freshwaters, land use and groundwater management strategies have been shown to affect groundwater quantity and quality (Lerner & Harris, 2009). Especially in areas of intensive agriculture and urbanization, groundwater is increasingly affected by contamination and overextraction (Burri et al., 2019; Dasgupta & Sanyal, 2022; Scanlon et al., 2007). As a result, there is growing consensus to enhance sustainable development, management, and governance of groundwater resources across the world (United Nations, 2022).

While groundwater has predominantly been studied and managed for its function as a freshwater resource, our understanding of groundwater as an ecosystem and its biotic communities remains limited (Mammola et al., 2019). To date, groundwater remains among the least documented ecosystems worldwide, mainly because of missing direct habitat accessibility and additional impediments (Ficetola et al., 2019; Mammola et al., 2021; Zagmajster et al., 2010). Nevertheless, the roles of groundwater as both a freshwater resource and an ecosystem are not entirely independent. For example, groundwater quality can be linked to groundwater organisms' contribution to nutrient cycling, contaminant degradation, and hydraulic connectivity (Boulton et al., 2008; Griebler & Avramov, 2015). Groundwater communities are unique; they consist, to a large extent, of highly adapted and small‐ranged, obligate groundwater organisms. As such, they might be particularly impacted by spatial flows across ecosystems. There is no photosynthetic primary production in groundwater and so groundwater organisms highly depend on allochthonous nutrient inflows (Fišer et al., 2014; Gibert et al., 1994; Humphreys, 2006). Simultaneously, they are vulnerable to contaminants from agricultural and urban areas (Becher et al., 2022; Castaño‐Sánchez et al., 2020; Di Lorenzo et al., 2014; Español et al., 2017).

Nanni et al. (2023) identified habitat change and climate change as the most pressing threats to subterranean ecosystems. At the same time, a notable gap exists in assessing the linkage between land use and groundwater communities (Mammola et al., 2020). Only a few studies have assessed possible correlations between surface land use and subterranean biodiversity (e.g., Cardoso et al., 2022; Couton et al., 2023; Español et al., 2017), and the spatial extent of such correlations, especially in the groundwater realm, remains even more unknown. Understanding the spatial scale is urgently needed, as multiple challenges still prevent today's management strategies from effectively protecting groundwater biodiversity (Huggins et al., 2023; Mammola et al., 2024).

Here, we evaluated correlations between surface land use (type and intensity) and associated effects on groundwater chemical quality and presence of specific groundwater fauna. Further, we investigated at what spatial scale relevant signals of surface groundwater linkages are apparent. To date, the deficiency of standardized, systematic, and highly resolved datasets has strongly limited assessments of land use correlations on groundwater biodiversity. Here, we used extensive data across the Swiss plateau, a region of variable but also pronounced high human population density and intensive agricultural land use. Groundwater amphipods were collected as part of a large citizen science program, where hundreds of municipal drinking water providers systematically sampled groundwater fauna from passively extracted water from shallow aquifers. We investigated (1) where drinking water extraction sites are located in respect to regional arrangement and distribution of land use cover; (2) how land use type and intensity correlates with local groundwater quality; (3) whether occurrence of specific groundwater fauna elements, such as amphipods, is correlated with surface land use type and intensity; and (4) at which spatial scales this interlinkage is most pronounced. Our findings not only demonstrate such a direct correlation of surface land use type and intensity on groundwater quality and occurrence of characteristic groundwater fauna, but also that these effects are apparent beyond current spatial extents of groundwater protection zones. Thus, currently applied regulations on land use may not be sufficient to protect groundwater resources from both a chemical and biological perspective, and a more integrated perspective—specifically considering cross‐ecosystem effects on human activities on groundwater integrity—may need to be applied to protect groundwater not only for human resource use but also as an ecosystem.

## MATERIALS AND METHODS

### Study area and focal taxa

**FIGURE 1:**
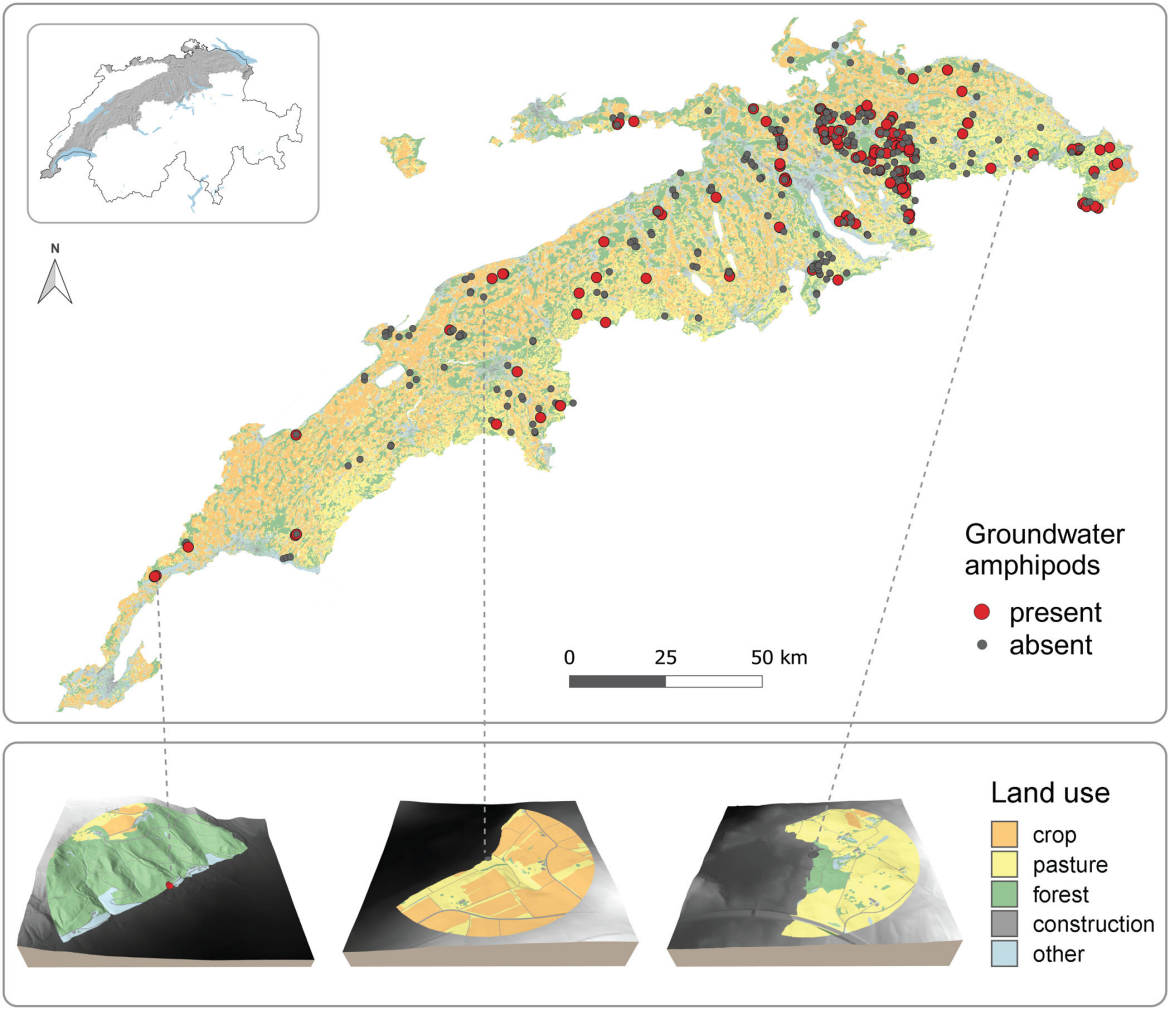
Sampling design across the Swiss plateau. Upper panel: Sampling sites with (red) and without (dark gray) groundwater amphipods across the Swiss plateau. Lower panel: Land use visualization of three sampling sites, extracted for a 600‐m buffer approximating the catchment area. Land use types are classified into crop (orange), pasture (yellow), forest (green), construction (gray), and remaining (blue) land use types.

We used data on groundwater amphipod occurrences across the Swiss plateau in central Europe. The study area spans 11,300 km^2^ and is located along a large molasse basin in the northern Alpine forelands. We focused on this region as it is a well‐defined biogeographic unit (BAFU, 2022) with relatively constant topography. The area is overall relatively densely populated and includes urban, agricultural, and forested ecosystem types with various land use intensities, arranged in a mosaic landscape structure (Figure 1). The sampling sites cover an elevation range of 359–1290 m above sea level. The data were collected as part of an extensive citizen science approach, where groundwater fauna samples were retrieved by hundreds of municipal drinking water providers (Knüsel et al., 2024c). Here, we focused on groundwater amphipods, which are the most common stygobiotic macroinvertebrates in Switzerland (Schneider et al., 2023). Groundwater amphipods were morphologically identified to the genus level (*Niphargus* and *Crangonyx*; Altermatt et al., 2019). The dataset consisted of 484 sampling sites (162 sites with and 322 sites without groundwater amphipods present; Figure 1).

### Land use data

We used highly resolved, vectorized spatial data on land use from Price et al. (2021) and elevation data with a spatial resolution of 2 m from the swissALTI3D digital elevation model (swisstopo, 2019). For each sampling site, we extracted land use data from a combination of multiple buffer sizes (between 10 m and 5000 m) with two buffer types (round and catchment). Specifically, we used buffer sizes of radii 10, 20, 50, 100, 200, 400, 600, 800, 1000, 2000, 3000, 4000, and 5000 m around each site. Round buffers were created using the given buffer sizes as radii around the sampling sites. Catchment buffers were constructed using a subset of the corresponding round buffer to approximate the drainage area (Figure 1). We included all surrounding land in the catchment buffer that was higher in elevation than the sampling site itself (5 m of elevation were subtracted from the sampling sites' elevation to avoid local, small‐scale surface maxima). All areas that were higher in elevation but not directly adjacent to the sampling site (threshold 50 m) were discarded, assuming they do not drain toward the sampling site. This method is an approximation of groundwater flow, which often but not always aligns with surface topography and further depends on factors like geology, groundwater table, and recharge (Rinderer et al., 2014; Tóth, 1963). For this reason we included two buffer types: a set of unrestricted, round buffers and a set of topography‐restricted catchment buffers. Finally, for each of the created buffers, we extracted data on forest, intensive pasture (hereafter pasture), crop, and construction land use proportions (Figure 1). All remaining categories were combined to “other” (see Appendix S1: Table S1 for a detailed classification documentation).

### Groundwater protection zone quantification

In Switzerland, groundwater protection zones are the most important instrument for resource‐use‐oriented groundwater quality preservation (BUWAL, 2004). They cover the drinking water extraction points and their immediate surroundings, in order to prevent the groundwater from impairment immediately before it is extracted. Data were retrieved from BAFU (2023), using zones S1, S2, and S3 (see BUWAL, 2004) that are, to date, in effect. These zones are usually determined by an expert and the assessment is tailored to the prevailing local hydrogeological conditions. The distance from the water extraction point to the outer edge of the largest zone (S3) generally spans not more than a few hundred meters. Specifically, zone S1 covers the immediate catchment location (ca. 10 m buffer around the extraction site). It is enclosed by zone S2 which is dimensioned by the residence time (residence of groundwater from the outer edge of S2 to the groundwater extraction point ≥10 days) and by distance (S1 to the outer edge of S2 in the direction of inflow ≥100 m). The largest zone, namely S3, is defined by distance (distance in the direction of inflow between outer edges of S2 and S3 ≈ distance between S1 and S2, and minimum downstream distance set by lower culmination point so that the water does not flow back in unfavorable conditions). Overall, 361 of our sampling sites could be associated with protection zones. For these, we extracted the maximum extent from the sampling site to the boundary of the largest protection zone (generally zone S3).

### Statistical analyses

We first investigated whether the observed land use proportions at the sampling sites differed from a null expectation. As a comparison with the sampled sites, we extracted land use data (using the same method as described above) for 484 randomly distributed sites across the Swiss plateau, but kept the proportion of sites from the Töss catchment constant, to match the higher density of sampling sites in this area. We used Wilcoxon tests with Bonferroni‐adjusted *p*‐values to compare the cumulated land use proportions between random and sampling sites. Therefore, land use data from 600 m catchment buffers were used (for both random and sampling sites), as for this spatial scale and buffer‐type combination, the highest values of McFadden's pseudo *R* ^2^ were found (see results below, “Spatial scale of land use effects on groundwater fauna”).

In the further statistical analyses, we included the three major land use types—forest, pasture, and crop, respectively. We tested for a relationship between the measured nitrate concentrations at 149 of the sampled spring catchment boxes and the respective land use proportions from 600‐m catchment buffers. Nitrate concentrations were measured by the water providers as part of their standard water quality self‐control assessments and these spot measurements generally reflect long‐term conditions. If multiple spot measurements were available from different inlets within the sampling site, we averaged the measurements. If multiple measurements were available across time, we used the one closest to the sampling period. We fit spatial regression models (R package “spaMM”; Rousset & Ferdy, 2014), including a spatially correlated random effect with Matérn covariance (based on the sampling sites' coordinates). We fit one model for each of the three land use types as fixed effects separately (forest, pasture, crop) and used a Gamma distribution with log‐link function to model the response variable (nitrate concentrations). Model fit was verified based on the visual inspection of diagnostic plots.

To assess the spatial scale of land use signals on groundwater fauna, we used a generalized linear model for each combination of buffer size, buffer type, and land use type. Groundwater amphipod presence/absence was used as response variable (binomial response distribution with logit link function) and the percentage coverage of a given land use type as explanatory variable. We extracted the slope estimate and calculated McFadden's pseudo *R* ^2^ (McFadden, 1974) by $1-\left(\text{deviance}/\text{null deviance}\right)$ as a goodness‐of‐fit measurement. Uncertainties of the pseudo *R* ^2^ were estimated by randomly subsampling 70% of the included sampling sites 1000 times and extracting the SD of McFadden's pseudo *R* ^2^ across all subsampling models. The pseudo *R* ^2^ values across the different buffer sizes (as a proxy for land use effects on groundwater amphipods, using the mean and SD of the mean among combinations of land use types and buffer types computed above) were then compared with the groundwater protection zone sizes.

Sites were visualized on a ternary plot based on their proportional forest, pasture, and crop coverage from the 600‐m catchment buffers, differentiating between sites with and without groundwater amphipods. A final spatial regression model was constructed to analyze groundwater amphipod presence/absence in response to land use and additional environmental covariates (see below) that have previously been used by Knüsel et al. (2024c) for modeling species‐level groundwater amphipod distributions. We used a binomial distribution with logit link to model the amphipod presence/absence. Land use was included as a single explanatory variable, representing the forest–agriculture (pasture and crop) gradient, which corresponds to the vertical axis of the ternary plot. Thereby, the combined contribution of the three land use types was considered as 100%. Additional environmental covariates included altitude, closest distances to any karstic and unconsolidated aquifers (BAFU, 2017), glaciation during the last glacial maximum (binary; Bini et al., 2009), and glacier cover time during the last glacial cycle (120–0 ka BP; Seguinot et al., 2018) (see Knüsel et al., 2024c for a detailed description of the covariates). The spatial regression model was fit using the R package “spaMM” (Rousset & Ferdy, 2014), including a spatially correlated random effect with Matérn covariance. To select the most parsimonious model based on the explanatory variables of the full model, we applied a stepwise backward model selection process based on AIC (R package “MASS”; Ripley et al., 2013).

## RESULTS

### Land use composition at drinking water extraction sites

**FIGURE 2:**
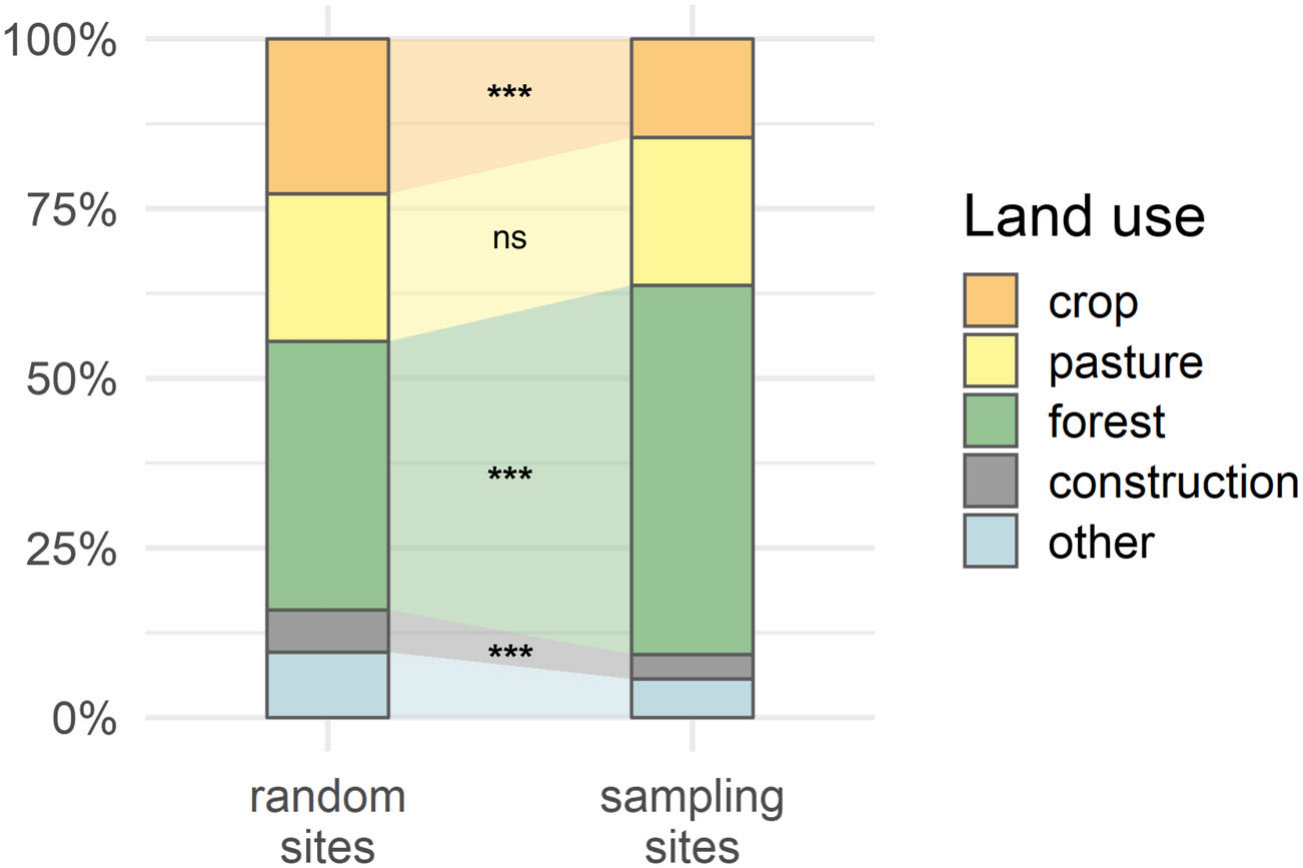
Comparison of land use type contributions between random and sampling sites. Data for random sites were extracted from 484 arbitrarily set locations across the Swiss plateau. Percentages refer to the cumulated percentages of land use coverage per type across all sites of the given class (random and sampling), using a 600‐m catchment buffer. The significance level was computed based on a Wilcoxon test with Bonferroni correction (*** for *p* < 0.001, ns for *p* ≥ 0.05).

Sampling sites and their immediate surroundings had significantly different land use compositions than randomly distributed sites across the study area (Figure 2 and Appendix S1: Figure S1). Specifically, sampling sites contained higher proportions of forest (Wilcoxon test with Bonferroni correction, *p* < 0.001) and lower proportions of crop (*p* < 0.001) and construction (*p* < 0.001) than randomly distributed sites. We did not find any difference in pasture coverage between sampling and random sites (*p* = 0.98).

### Land use‐dependent nitrogen concentrations

**FIGURE 3:**
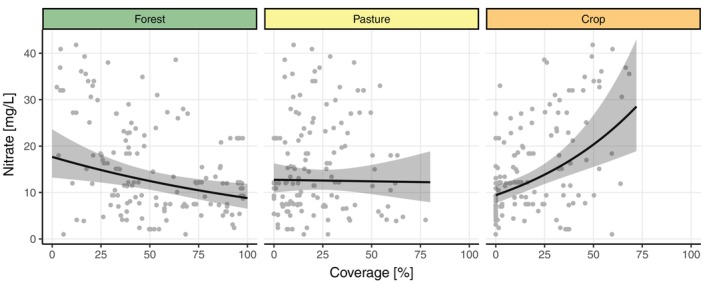
Correlations between land use type proportions and measured nitrate concentration. Nitrate concentrations were measured in the groundwater at the sampling sites. Regression lines correspond to a spatial regression model with 95% confidence intervals of the estimated mean (see also Appendix S1: Table S2).

We found a significant negative relationship between forest coverage within the immediate catchment area and nitrate concentrations measured in the groundwater (−0.0070, 95% CI −0.012, −0.002), with nitrate concentrations decreasing with increasing forest coverage (Figure 3 and Appendix S1: Table S2). An opposite effect was found for crop, where nitrate concentrations increased with increasing crop coverage (0.015, 95% CI 0.008, 0.023, Figure 3). No significant relationship was detected for pasture (−0.0005, 95% CI −0.008, 0.007). Spatial autocorrelation was identified up to around a 2‐km distance between sampling sites (Appendix S1: Figure S2).

### Spatial scale of land use effects on groundwater fauna

**FIGURE 4:**
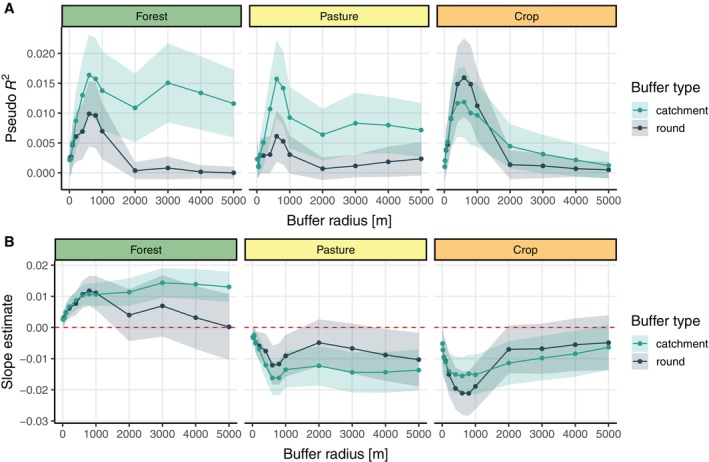
Spatial scale of land use composition effects on groundwater fauna. For each combination of land use type, buffer size, and buffer type, one generalized linear model was built using groundwater amphipod presence/absence as response. McFadden's pseudo *R* ^2^ with corresponding SDs (A) and the estimated slope of the land use coverage effect with SEs (B) are plotted.

**FIGURE 5:**
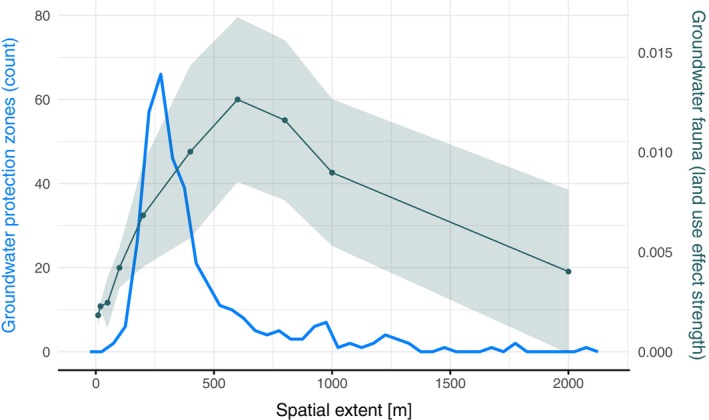
Spatial scale of land use composition effects on groundwater fauna compared with groundwater protection zone sizes for drinking water extraction. Effect strength on groundwater fauna is shown as the mean and SD of the mean pseudo *R* ^2^ values across the land use and buffer types plotted in Figure 4A. The spatial extent of the groundwater protection zones was extracted based on the maximal distance from the sampling site to the edge of the protected zone (*n* = 361).

When assessing the spatial scale of land use signals on groundwater fauna, we found highest pseudo *R* ^2^ values at buffer sizes of around 400–1000 m, peaking at 600 m (Figure 4). This pattern was consistent across all land use types investigated, and also consistent for both round and catchment buffer types. For forest and pasture land use, we found higher pseudo *R* ^2^ values when using catchment buffers than when using round buffers. Crop coverage showed a less distinctive pattern, likely because there is, a priori, a generally low percentage of crop in the immediate catchment of the drinking water extraction sites. Slope estimates, approximating the direction of the effect of a given land use type on groundwater amphipod presence/absence were positive for forest and negative for pasture and crop, respectively (Figure 4). Groundwater protection zones were found to have a median extent of 327 m from the sampling site. The spatial scale of land use effects on groundwater fauna is thus 1.2‐ to 3‐fold larger than the average extent of the groundwater protection zones (Figure 5). Of the included sampling sites, 67% had a protection zone smaller than 400 m in extent.

### Groundwater amphipod occurrence under different environmental conditions

**FIGURE 6:**
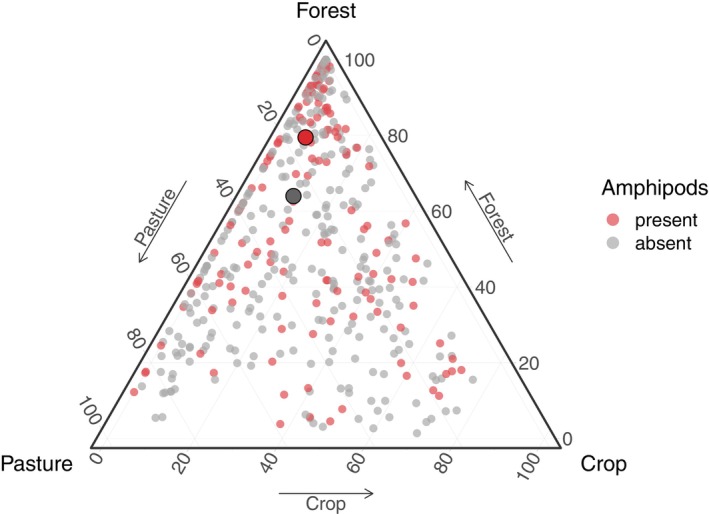
Ternary plot using the contribution of the main land use types to each sampling site. Sampling sites are grouped into sites with (red) and without (gray) amphipods. Large points show the mean values for both groups.

When comparing sites with and without groundwater amphipods on a ternary plot, including forest, pasture, and crop as axes, we found sites with presence of amphipods to have higher forest but lower pasture and crop composition than sites where no amphipods were found (Figure 6). This result was further supported by a final spatial regression model in which we fit groundwater amphipod presence/absence in response to land use and additional broad environmental covariates. The latter were previously used by Knüsel et al. (2024c) to model regional groundwater amphipod species' distributions without assessing anthropogenic factors. After applying a stepwise backward model selection, the most parsimonious model contained land use (0.013, 95% CI 0.0038, 0.023) and altitude (0.0032, 95% CI 0.00095, 0.0057) as explanatory variables and a spatially correlated random effect (Table 1). Land use had the lowest *p*‐value of all explanatory variables. Thus, higher forest—and, conversely, lower pasture and crop coverage—within about a 600‐m drainage area of the sampling sites were estimated to have a positive association with groundwater amphipod occurrence. Covariates related to hydrogeology and historic glaciation were not retained in the most parsimonious model. Spatial autocorrelation in the residuals of the model was estimated up to an 8‐km distance between sampling sites.

**TABLE 1:** Results of the most parsimonious spaMM spatial mixed‐effect model (binomial family with logit‐link) for associations between environmental covariates and groundwater amphipod occurrence.

	Groundwater amphipod occurrence				
	Est	SE	*t*	95% CI	*p* value
Predictors					
Intercept	3.76	0.75	−4.99		<0.001
Land use	0.013	0.0045	2.88	0.0038, 0.023	0.0042
Altitude	0.0032	0.0011	2.88	0.00095, 0.0057	0.0041
Random effect					
ν, ρ	1.26, 0.00061				
Random effect var. λ	1.32				
*N*_Obs_	484				

## DISCUSSION

Substantial research documents land use effects on surface freshwater ecosystems, including how land use intensifications (Collyer et al., 2023), land use types (Cereghetti & Altermatt, 2023; Kaelin & Altermatt, 2016; Petsch et al., 2021), or land use associated run‐offs (Weijters et al., 2009) affect the structure, occurrence, and diversity of aquatic organisms. Contrastingly, much less is known about land use impacts on groundwater communities and the spatial extent of such impacts, despite postulated linkages and resource flows between surface and groundwater systems (Saccò et al., 2024). Analyzing extensive data on groundwater amphipod occurrence from hundreds of drinking water extraction sites, we found a clear signal of land use on amphipod occurrence, with forest indicating a positive and agricultural land use a negative effect. In addition, a spatial extent of 400–1000 m was identified to be most relevant in shaping this correlation, indicating that the fauna of shallow groundwater aquifers is not only shaped by land use effects of the immediate surroundings. Instead, land use up to mid‐distance proximity to the sampling site should be included. Such a link between aboveground land use and groundwater biodiversity is crucial for biodiversity conservation and requires respective and adequate management of groundwater ecosystems.

Only few, and mostly localized, studies have found indications of land use effects on groundwater macroinvertebrates (Couton et al., 2023; Dole‐Olivier et al., 2009; Koch et al., 2021; Korbel et al., 2013). Here, studying a large and varyingly managed landscape, we found land use to have a stronger effect on groundwater amphipod occurrence than any of the other environmental covariates that were included in the spatial regression model. While geologic, topographic, and historic factors might play an important role at the species‐level distributions (Knüsel et al., 2024b, 2024c), we found indications that land use might affect groundwater communities at a more fundamental level. At the drinking water extraction sites sampled in this study, especially the gradient between forest and agricultural land use showed patterns in groundwater amphipod occurrence. We speculate that these patterns could be even more pronounced if samples from groundwater aquifers unsuitable for drinking water extraction, that is, from highly urbanized (see Koch et al., 2021) or intense agricultural areas were also considered. Local hydrogeological conditions (such as porosity) might also influence point‐specific groundwater fauna occurrence (see Korbel et al., 2019), but this could not be assessed due to missing high‐resolution hydrogeological data (which is a common limitation in groundwater ecology).

All of our sampling sites were established by municipal water providers to capture groundwater for drinking water usage. Consequently, all of these sites were likely a priori expected to be of good to very good water quality, as the extracted groundwater has to meet quality criteria for drinking water use. This also suggests that the study sites may be, in general, of low (or lower than average) exposure to pollutants such as pesticides, fertilizers, and substances from urban and industrial runoffs, to maintain high water quality. Indeed, the sampled groundwater catchment sites were situated at locations with higher forest and lower crop and construction coverage in the catchment area than randomly placed sites across the study area. We can thus assume that the study sites are, on average, less anthropogenically affected than a random groundwater site. Nevertheless, we still found a clear correlation between measured nitrate concentrations at the sampling sites and the amount of crop or forest coverage in the drainage area. Sites with more crop coverage had higher nitrate concentrations in the groundwater, whereas sites with more forest coverage had lower nitrate concentrations. Our findings are in line with observations from the national groundwater monitoring program (BAFU, 2019) and previous studies (Lerner & Harris, 2009 and references therein), consistent also with trends of other contaminants such as pesticides (BAFU, 2019).

Our analysis revealed first insights into the spatial scale of land use effects on groundwater fauna. We observed that models incorporating land use data from within a spatial range of 400–1000 m (peaking at 600 m) around the sampling sites had the highest *R* ^2^ values than models covering smaller or larger spatial scales. Comparable spatial scales of land‐water biodiversity linkages have been identified for aboveground systems within the same biogeographic area (Zhang et al., 2023). Also, the spatial scale, as identified in this study, falls within the range observed in studies assessing the effects of land use on groundwater quality across different geographic regions (Johnson & Belitz, 2009 and references therein; Gallagher & Gergel, 2017). Spatial patterns of land use effects on groundwater quality and hydrochemical parameters might thus align with local groundwater fauna composition. The spatial extent of this effect may depend on factors such as topology, aquifer composition and depth of the water extraction (see, e.g., Yan et al., 2022), and thus may not be directly transferred to any other groundwater systems, yet the general conclusion of a more regional effect of land use and agricultural practices on groundwater fauna may hold generally true.

Interestingly, the here identified spatial scale largely exceeds the extent of governmentally defined groundwater protection zones (BUWAL, 2004) for most of the sampling sites (on average by 1.2‐ to 3‐fold). These “groundwater protection zones” are defined to minimize contamination and negative quality effects on the groundwater due to aboveground human activities. Here, we show that observed signals on terrestrial land use are extending well beyond these zones with associated effects in the groundwater detected. We thus conclude that the currently implemented protection zones (designated to ensure high water quality at the extraction sites) might not be large enough to prevent the negative effects of land use on groundwater communities. Possibly, the spatial scale detected by us may reflect the actual inflow area (area of contribution, Zuströmbereich Z_u_; BUWAL, 2004), defining the actual origin and residence of the water extracted, which is not captured by the more narrowly implemented groundwater protection zones. If groundwater ecosystems are indeed to be protected from harmful land use impacts, an appropriate spatial extent must be considered for the realization of management and conservation actions, and an adequate definition and management of buffer zones would need to be defined for drinking water catchment sites in Switzerland.

Given the tight linkage between shallow groundwater, soil, and surface ecosystems (Schneider et al., 2023), understanding cross‐ecosystem effects on groundwater communities is crucial. This need is even more pressing given the increasing anthropogenic pressures on groundwater ecosystems, such as water and geothermal energy extraction, contamination, climate change, and habitat degradation (Mammola et al., 2019). We here identified signals of land use on groundwater organisms that exceed the spatial scale of present‐day drinking water protection zones. We postulate that incorporating an ecosystem perspective into groundwater management strategies will ultimately enhance the effectiveness of protecting both groundwater quality and biodiversity.

## AUTHOR CONTRIBUTIONS

Mara Knüsel: conceptualization, methodology, formal analysis, visualization, original draft writing, review and editing. Roman Alther and Florian Altermatt: conceptualization, funding acquisition, methodology, supervision, review and editing.

## CONFLICT OF INTEREST STATEMENT

The authors declare no conflicts of interest.

## Supporting information


Appendix S1.

## Data Availability

Data (Knüsel et al., 2024a) are available in Zenodo at https://doi.org/10.5281/zenodo.12731054. Environmental data in addition to land use are available from Knüsel et al. (2024d) at https://doi.org/10.5281/zenodo.12570591.

## References

[eap3040-bib-0001] Allan, J. D. 2004. “Landscapes and Riverscapes: The Influence of Land Use on Stream Ecosystems.” Annual Review of Ecology, Evolution, and Systematics 35(1): 257–284. 10.1146/annurev.ecolsys.35.120202.110122.

[eap3040-bib-0002] Altermatt, F. , R. Alther , C. Fišer , and V. Švara . 2019. “Amphipoda (Flohkrebse) Der Schweiz: Checkliste, Bestimmung Und Atlas.” In Fauna Helvetica, Vol. 32. Neuchâtel: info fauna, Centre suisse de cartographie de la faune.

[eap3040-bib-0003] BAFU . 2017. Grundwasserkörper Der Schweiz. Bern: Bundesamt für Umwelt BAFU http://data.geo.admin.ch/ch.bafu.grundwasserkoerper/data.zip.

[eap3040-bib-0004] BAFU . 2019. Zustand Und Entwicklung Grundwasser Schweiz. Ergebnisse Der Nationalen Grundwasserbeobachtung NAQUA, Stand 2016. Bern: Umwelt‐Zustand Nr. 1901. Bundesamt für Umwelt BAFU.

[eap3040-bib-0005] BAFU . 2022. Die Biogeografischen Regionen Der Schweiz. 1. Aktualisierte Auflage 2022. Erstausgabe 2001. Bern: Umwelt‐Wissen Nr. 2214. Bundesamt für Umwelt BAFU.

[eap3040-bib-0006] BAFU . 2023. Planerischer Gewässerschutz: Grundwasserschutzzonen, ‐Areale Und Gewässerschutzbereiche. Bern: V1.2. Bundesamt für Umwelt BAFU https://www.geodienste.ch/services/planerischer_gewaesserschutz.

[eap3040-bib-0007] Becher, J. , C. Englisch , C. Griebler , and P. Bayer . 2022. “Groundwater Fauna Downtown – Drivers, Impacts and Implications for Subsurface Ecosystems in Urban Areas.” Journal of Contaminant Hydrology 248: 104021. 10.1016/j.jconhyd.2022.104021. 35605354

[eap3040-bib-0008] Bini, A. , J.‐F. Buoncristiani , S. Couterrand , D. Ellwanger , M. Felber , D. Florineth , H. Graf , et al. 2009. Die Schweiz Während Des Letzteiszeitlichen Maximums (LGM): 1:500 000. Wabern: Bundesamt für Landestopografie swisstopo.

[eap3040-bib-0009] Boulton, A. J. , G. D. Fenwick , P. J. Hancock , and M. S. Harvey . 2008. “Biodiversity, Functional Roles and Ecosystem Services of Groundwater Invertebrates.” Invertebrate Systematics 22(2): 103. 10.1071/IS07024.

[eap3040-bib-0010] Burdon, F. J. , N. A. Munz , M. Reyes , A. Focks , A. Joss , K. Räsänen , F. Altermatt , R. I. L. Eggen , and C. Stamm . 2019. “Agriculture Versus Wastewater Pollution as Drivers of Macroinvertebrate Community Structure in Streams.” The Science of the Total Environment 659: 1256–1265. 10.1016/j.scitotenv.2018.12.372. 31096338

[eap3040-bib-0011] Burri, N. M. , R. Weatherl , C. Moeck , and M. Schirmer . 2019. “A Review of Threats to Groundwater Quality in the Anthropocene.” The Science of the Total Environment 684: 136–154. 10.1016/j.scitotenv.2019.05.236. 31153063

[eap3040-bib-0012] BUWAL . 2004. Wegleitung Grundwasserschutz. Bern: Vollzug Umwelt. Bundesamt für Umwelt, Wald und Landschaft BUWAL https://www.bafu.admin.ch/bafu/de/home/themen/wasser/publikationen‐studien/publikationen‐wasser/wegleitung‐grundwasserschutz.html.

[eap3040-bib-0013] Cardoso, R. C. , R. L. Ferreira , and M. Souza‐Silva . 2022. “Multi‐Spatial Analysis on Cave Ecosystems to Predict the Diversity of Subterranean Invertebrates.” Basic and Applied Ecology 65: 111–122. 10.1016/j.baae.2022.11.007.

[eap3040-bib-0014] Castaño‐Sánchez, A. , G. C. Hose , and A. S. P. Reboleira . 2020. “Ecotoxicological Effects of Anthropogenic Stressors in Subterranean Organisms: A Review.” Chemosphere 244: 125422. 10.1016/j.chemosphere.2019.125422. 31805461

[eap3040-bib-0015] Cereghetti, E. , and F. Altermatt . 2023. “Spatiotemporal Dynamics in Freshwater Amphipod Assemblages Are Associated with Surrounding Terrestrial Land Use Type.” Ecosphere 14(3): e4469. 10.1002/ecs2.4469.

[eap3040-bib-0016] Collyer, G. , D. M. Perkins , D. K. Petsch , T. Siqueira , and V. Saito . 2023. “Land‐Use Intensification Systematically Alters the Size Structure of Aquatic Communities in the Neotropics.” Global Change Biology 29(14): 4094–4106. 10.1111/gcb.16720. 37059700

[eap3040-bib-0017] Couton, M. , S. Hürlemann , A. Studer , R. Alther , and F. Altermatt . 2023. “Groundwater Environmental DNA Metabarcoding Reveals Hidden Diversity and Reflects Land‐Use and Geology.” Molecular Ecology 32(13): 3497–3512. 10.1111/mec.16955. 37067032

[eap3040-bib-0018] Dasgupta, B. , and P. Sanyal . 2022. “Linking Land Use Land Cover Change to Global Groundwater Storage.” The Science of the Total Environment 853: 158618. 10.1016/j.scitotenv.2022.158618. 36084786

[eap3040-bib-0019] Deharveng, L. , F. Stoch , J. Gibert , A. Bedos , D. Galassi , M. Zagmajster , A. Brancelj , et al. 2009. “Groundwater Biodiversity in Europe.” Freshwater Biology 54(4): 709–726. 10.1111/j.1365-2427.2008.01972.x.

[eap3040-bib-0020] Di Lorenzo, T. , W. D. Di Marzio , M. E. Sáenz , M. Baratti , A. A. Dedonno , A. Iannucci , S. Cannicci , G. Messana , and D. M. P. Galassi . 2014. “Sensitivity of Hypogean and Epigean Freshwater Copepods to Agricultural Pollutants.” Environmental Science and Pollution Research International 21(6): 4643–4655. 10.1007/s11356-013-2390-6. 24352541

[eap3040-bib-0021] Dole‐Olivier, M.‐J. , F. Malard , D. Martin , T. Lefébure , and J. Gibert . 2009. “Relationships between Environmental Variables and Groundwater Biodiversity at the Regional Scale.” Freshwater Biology 54(4): 797–813. 10.1111/j.1365-2427.2009.02184.x.

[eap3040-bib-0022] Español, C. , F. A. Comín , B. Gallardo , J. Yao , J. L. Yela , F. Carranza , A. Zabaleta , et al. 2017. “Does Land Use Impact on Groundwater Invertebrate Diversity and Functionality in Floodplains?” Ecological Engineering 103: 394–403. 10.1016/j.ecoleng.2016.11.061.

[eap3040-bib-0023] Ficetola, G. F. , C. Canedoli , and F. Stoch . 2019. “The Racovitzan Impediment and the Hidden Biodiversity of Unexplored Environments.” Conservation Biology 33(1): 214–216. 10.1111/cobi.13179. 29962096

[eap3040-bib-0024] Fišer, C. , T. Pipan , and D. C. Culver . 2014. “The Vertical Extent of Groundwater Metazoans: An Ecological and Evolutionary Perspective.” Bioscience 64(11): 971–979. 10.1093/biosci/biu148.

[eap3040-bib-0025] Gallagher, T. L. , and S. E. Gergel . 2017. “Landscape Indicators of Groundwater Nitrate Concentrations: An Approach for Trans‐Border Aquifer Monitoring.” Ecosphere 8(12): e02047. 10.1002/ecs2.2047.

[eap3040-bib-0026] Gibert, J. , D. Danielopol , and J. A. Stanford . 1994. Groundwater Ecology. Aquatic Biology [Sic] Series. San Diego: Academic Press.

[eap3040-bib-0027] Giordano, M. 2009. “Global Groundwater? Issues and Solutions.” Annual Review of Environment and Resources 34(1): 153–178. 10.1146/annurev.environ.030308.100251.

[eap3040-bib-0028] Gleeson, T. , K. M. Befus , S. Jasechko , E. Luijendijk , and M. B. Cardenas . 2016. “The Global Volume and Distribution of Modern Groundwater.” Nature Geoscience 9(2): 161–167. 10.1038/ngeo2590.

[eap3040-bib-0029] Gounand, I. , E. Harvey , C. J. Little , and F. Altermatt . 2018. “Meta‐Ecosystems 2.0: Rooting the Theory into the Field.” Trends in Ecology & Evolution 33(1): 36–46. 10.1016/j.tree.2017.10.006. 29102408

[eap3040-bib-0030] Griebler, C. , and M. Avramov . 2015. “Groundwater Ecosystem Services: A Review.” Freshwater Science 34(1): 355–367. 10.1086/679903.

[eap3040-bib-0031] Huggins, X. , T. Gleeson , D. Serrano , S. Zipper , F. Jehn , M. M. Rohde , R. Abell , K. Vigerstol , and A. Hartmann . 2023. “Overlooked Risks and Opportunities in Groundwatersheds of the World's Protected Areas.” Nature Sustainability 6(7): 855–864. 10.1038/s41893-023-01086-9.

[eap3040-bib-0032] Humphreys, W. F. 2006. “Aquifers: The Ultimate Groundwater‐Dependent Ecosystems.” Australian Journal of Botany 54(2): 115. 10.1071/BT04151.

[eap3040-bib-0033] Johnson, T. D. , and K. Belitz . 2009. “Assigning Land Use to Supply Wells for the Statistical Characterization of Regional Groundwater Quality: Correlating Urban Land Use and VOC Occurrence.” Journal of Hydrology 370(1–4): 100–108. 10.1016/j.jhydrol.2009.02.056.

[eap3040-bib-0034] Kaelin, K. , and F. Altermatt . 2016. “Landscape‐Level Predictions of Diversity in River Networks Reveal Opposing Patterns for Different Groups of Macroinvertebrates.” Aquatic Ecology 50(2): 283–295. 10.1007/s10452-016-9576-1.

[eap3040-bib-0035] Kirschner, D. , Y. Zhang , W. Zhong , X. Zhang , L. Pellissier , and F. Altermatt . 2023. “Homogeneity in Terrestrial Land Cover Is Reflected in Fish Diversity Patterns in a Chinese River System.” Environmental DNA 5(6): 1679–1690. 10.1002/edn3.480.

[eap3040-bib-0036] Kløve, B. , P. Ala‐aho , G. Bertrand , Z. Boukalova , A. Ertürk , N. Goldscheider , J. Ilmonen , et al. 2011. “Groundwater Dependent Ecosystems. Part I: Hydroecological Status and Trends.” Environmental Science & Policy 14(7): 770–781. 10.1016/j.envsci.2011.04.002.

[eap3040-bib-0037] Knight, T. M. , M. W. McCoy , J. M. Chase , K. A. McCoy , and R. D. Holt . 2005. “Trophic Cascades across Ecosystems.” Nature 437(7060): 880–883. 10.1038/nature03962. 16208370

[eap3040-bib-0038] Knüsel, M. , R. Alther , and F. Altermatt . 2024a. “Data for: Terrestrial Land Use Signals on Groundwater Fauna Beyond Current Protection Buffers (Ecological Applications, 2024) (v1.0) [Data Set].” Zenodo. 10.5281/zenodo.12731054. PMC11610645 39424409

[eap3040-bib-0039] Knüsel, M. , R. Alther , and F. Altermatt . 2024b. “Pronounced Changes of Subterranean Biodiversity Patterns along a Late Pleistocene Glaciation Gradient.” Ecography 2024: e07321. 10.1111/ecog.07321.

[eap3040-bib-0040] Knüsel, M. , R. Alther , N. Locher , A. Ozgul , C. Fišer , and F. Altermatt . 2024c. “Systematic and Highly Resolved Modelling of Biodiversity in Inherently Rare Groundwater Amphipods.” Journal of Biogeography. 10.1111/jbi.14975.

[eap3040-bib-0041] Knüsel, M. , R. Alther , N. Locher , A. Ozgul , C. Fišer , and F. Altermatt . 2024d. “Data for: Systematic and Highly Resolved Modelling of Biodiversity in Inherently Rare Groundwater Amphipods (Journal of Biogeography, 2024) [Data Set].” Journal of Biogeography (v1.0). Zenodo. 10.5281/zenodo.12570591.

[eap3040-bib-0042] Koch, F. , K. Menberg , S. Schweikert , C. Spengler , H. J. Hahn , and P. Blum . 2021. “Groundwater Fauna in an Urban Area – Natural or Affected?” Hydrology and Earth System Sciences 25(6): 3053–3070. 10.5194/hess-25-3053-2021.

[eap3040-bib-0043] Korbel, K. L. , P. J. Hancock , P. Serov , R. P. Lim , and G. C. Hose . 2013. “Groundwater Ecosystems Vary with Land Use across a Mixed Agricultural Landscape.” Journal of Environmental Quality 42(2): 380–390. 10.2134/jeq2012.0018. 23673830

[eap3040-bib-0044] Korbel, K. L. , S. Stephenson , and G. C. Hose . 2019. “Sediment Size Influences Habitat Selection and Use by Groundwater Macrofauna and Meiofauna.” Aquatic Sciences 81(2): 1–2. 10.1007/s00027-019-0636-1.

[eap3040-bib-0045] Lerner, D. N. , and B. Harris . 2009. “The Relationship between Land Use and Groundwater Resources and Quality.” Land Use Policy 26: S265–S273. 10.1016/j.landusepol.2009.09.005.

[eap3040-bib-0046] Mammola, S. , F. Altermatt , R. Alther , I. R. Amorim , R. I. Băncilă , P. A. V. Borges , T. Brad , et al. 2024. “Perspectives and Pitfalls in Preserving Subterranean Biodiversity through Protected Areas.” npj Biodiversity 3(1): 2. 10.1038/s44185-023-00035-1. PMC11332058 39242876

[eap3040-bib-0047] Mammola, S. , I. R. Amorim , M. E. Bichuette , P. A. V. Borges , N. Cheeptham , S. J. B. Cooper , D. C. Culver , et al. 2020. “Fundamental Research Questions in Subterranean Biology.” Biological Reviews 95: 1855–1872. 10.1111/brv.12642. 32841483

[eap3040-bib-0048] Mammola, S. , P. Cardoso , D. C. Culver , L. Deharveng , R. L. Ferreira , C. Fišer , D. M. P. Galassi , et al. 2019. “Scientists' Warning on the Conservation of Subterranean Ecosystems.” Bioscience 69(8): 641–650. 10.1093/biosci/biz064.

[eap3040-bib-0049] Mammola, S. , E. Lunghi , H. Bilandžija , P. Cardoso , V. Grimm , S. I. Schmidt , T. Hesselberg , and A. Martínez . 2021. “Collecting Eco‐Evolutionary Data in the Dark: Impediments to Subterranean Research and how to Overcome Them.” Ecology and Evolution 11(11): 5911–5926. 10.1002/ece3.7556. 34141192 PMC8207145

[eap3040-bib-0050] McFadden, D. 1974. “Conditional Logit Analysis of Qualitative Choice Behavior.” In Frontiers in Econometrics, edited by P. Zarembka , 105–142. New York, NY: Academic Press.

[eap3040-bib-0051] Munz, N. A. , F. J. Burdon , D. de Zwart , M. Junghans , L. Melo , M. Reyes , U. Schönenberger , et al. 2017. “Pesticides Drive Risk of Micropollutants in Wastewater‐Impacted Streams during Low Flow Conditions.” Water Research 110: 366–377. 10.1016/j.watres.2016.11.001. 27919541

[eap3040-bib-0052] Nanni, V. , E. Piano , P. Cardoso , M. Isaia , and S. Mammola . 2023. “An Expert‐Based Global Assessment of Threats and Conservation Measures for Subterranean Ecosystems.” Biological Conservation 283: 110136. 10.1016/j.biocon.2023.110136.

[eap3040-bib-0053] Petsch, D. K. , S. A. Blowes , A. S. Melo , and J. M. Chase . 2021. “A Synthesis of Land Use Impacts on Stream Biodiversity across Metrics and Scales.” Ecology 102(11): e03498. 10.1002/ecy.3498. 34314043

[eap3040-bib-0054] Price, B. , N. Huber , C. Ginzler , R. Pazúr , and M. Rüetschi . 2021. “The Habitat Map of Switzerland (V1).” EnviDat. 10.16904/envidat.262.

[eap3040-bib-0055] Rinderer, M. , H. J. van Meerveld , and J. Seibert . 2014. “Topographic Controls on Shallow Groundwater Levels in a Steep, Prealpine Catchment: When Are the TWI Assumptions Valid?” Water Resources Research 50(7): 6067–6080. 10.1002/2013WR015009.

[eap3040-bib-0056] Ripley, B. , B. Venables , D. M. Bates , K. Hornik , A. Gebhardt , D. Firth , and M. B. Ripley . 2013. “Package ‘MASS’.” Cran r 538: 113–120.

[eap3040-bib-0057] Rousset, F. , and J.‐B. Ferdy . 2014. “Testing Environmental and Genetic Effects in the Presence of Spatial Autocorrelation.” Ecography 37(8): 781–790. 10.1111/ecog.00566.

[eap3040-bib-0058] Saccò, M. , S. Mammola , F. Altermatt , R. Alther , R. Bolpagni , A. Brancelj , D. Brankovits , et al. 2024. “Groundwater is a Hidden Global Keystone Ecosystem.” Global Change Biology 30(1): e17066. 10.1111/gcb.17066. 38273563

[eap3040-bib-0059] Scanlon, B. R. , I. Jolly , M. Sophocleous , and L. Zhang . 2007. “Global Impacts of Conversions from Natural to Agricultural Ecosystems on Water Resources: Quantity Versus Quality.” Water Resources Research 43(3): W03437. 10.1029/2006WR005486.

[eap3040-bib-0060] Schneider, A. S. , M. Knüsel , and F. Altermatt . 2023. “Assessment of Occurrence, Diversity, and Biomass of Macroinvertebrates in Swiss Groundwater Systems Using Citizen Science Data.” Subterranean Biology 46: 147–164. 10.3897/subtbiol.46.112569.

[eap3040-bib-0061] Seguinot, J. , S. Ivy‐Ochs , G. Jouvet , M. Huss , M. Funk , and F. Preusser . 2018. “Modelling Last Glacial Cycle Ice Dynamics in the Alps.” The Cryosphere 12(10): 3265–3285. 10.5194/tc-12-3265-2018.

[eap3040-bib-0062] swisstopo . 2019. “SwissALTI3D.” Bundesamt für Landestopografie swisstopo. https://www.swisstopo.admin.ch/de/hoehenmodell-swissalti3d.

[eap3040-bib-0063] Tóth, J. 1963. “A Theoretical Analysis of Groundwater Flow in Small Drainage Basins.” Journal of Geophysical Research 68(16): 4795–4812. 10.1029/JZ068i016p04795.

[eap3040-bib-0064] United Nations . 2022. Groundwater: Making the Invisible Visible. The United Nations World Water Development Report 2022. Paris: UNESCO.

[eap3040-bib-0065] Weijters, M. J. , J. H. Janse , R. Alkemade , and J. T. A. Verhoeven . 2009. “Quantifying the Effect of Catchment Land Use and Water Nutrient Concentrations on Freshwater River and Stream Biodiversity.” Aquatic Conservation: Marine and Freshwater Ecosystems 19(1): 104–112. 10.1002/aqc.989.

[eap3040-bib-0066] Yan, J. , J. Chen , and W. Zhang . 2022. “Impact of Land Use and Cover on Shallow Groundwater Quality in Songyuan City, China: A Multivariate Statistical Analysis.” Environmental Pollution 307: 119532. 10.1016/j.envpol.2022.119532. 35636717

[eap3040-bib-0067] Zagmajster, M. , D. C. Culver , M. C. Christman , and B. Sket . 2010. “Evaluating the Sampling Bias in Pattern of Subterranean Species Richness: Combining Approaches.” Biodiversity and Conservation 19(11): 3035–3048. 10.1007/s10531-010-9873-2.

[eap3040-bib-0068] Zhang, H. , E. Mächler , F. Morsdorf , P. A. Niklaus , M. E. Schaepman , and F. Altermatt . 2023. “A Spatial Fingerprint of Land‐Water Linkage of Biodiversity Uncovered by Remote Sensing and Environmental DNA.” The Science of the Total Environment 867: 161365. 10.1016/j.scitotenv.2022.161365. 36634788

