# Supplementary material for: Terrestrial land use signals on groundwater fauna beyond current protection buffers
Source: Ecol Appl. 2024 Oct 18;34(8):e3040. doi: 10.1002/eap.3040 (PMC11610645; doi:10.1002/eap.3040)
Supplement: Supplementary file 1 — Appendix S1. [file EAP-34-e3040-s001.pdf]

Appendix S1

Supporting Information for
Terrestrial land use signals on groundwater fauna beyond current protection buffers

Mara Knüsel, Roman Alther, Florian Altermatt

Ecological Applications

Table S1. Categorization of land use types based on the data from Price et al. (2021).

Categorization of data from the Swiss Habitat Map (Price et al. 2021)		Categorization by the authors of the study: Crop	Pasture	Forest	Construction	Other
1 Gewässer						
	1.1 Stehende Gewässer					x
	1.2 Fliessgewässer					x
	1.3 Quellen und Quellfluren					x
	1.4 Unterirdische Gewässer					x
2 Ufer und der Feuchtgebiete						
	2.0 Künstliche Ufer					x
	2.1 Ufer mit Vegetation					x
	2.2 Flachmoore					x
	2.3 Feuchtwiesen					x
	2.4 Hochmoore					x
	2.5 Wechselfeuchte Pionierfluren					x
3 Gletscher, Fels, Schutt und Geröll						
	3.1 Gletscher, Firn- und Schneefleder					x
	3.2 Alluvionen und Moränen					x
	3.3 Steinschutt- und Geröllfluren					x
	3.4 Felsen					x
	3.5 Höhlen					x
4 Grünland (Naturrasen, Wiesen und Weiden)						
	4.0 Kunstrasen		x			
	4.1 Pionierfluren auf Felsböden (Felsgrusfluren)					x
	4.2 Wärmeliebende Trockenrasen					x
	4.3 Gebirgs-Magerrasen					x
	4.4 Schneetälchen					x
	4.5 Fettwiesen und -weiden		x			
	4.6 Grasbrachen					x
5 Krautsäume, Hochstaudenfluren und Gebüsche						
	5.1 Krautsäume					x
	5.2 Hochstauden- und Schlagfluren					x
	5.3 Gebüsche					x
	5.3 Gebüsche					x
	5.4 Zwergstrauchheiden					x
6 Wälder						
	6.0 Forstpflanzungen und Einzelbäume			x		
	6.1 Bruch- und Auenwälder			x		
	6.2 Buchenwälder			x		
	6.3 Andere Laubwälder			x		
	6.3a Schluchtwälder			x		
	6.3b Eichenwälder			x		
	6.3c Wärmeliebende Sekundärwälder			x		
	6.4 Wärmeliebende Föhrenwälder			x		
	6.5 Hochmoorwälder			x		
	6.6 Gebirgsnadelwälder			x		
7 Pioniervegetation gestörter Plätze (Ruderalstandorte)						
	7.1 Trittrasen und Ruderalfluren					x
	7.2 Anthropogene Steinfluren					x
8 Pflanzungen, Äcker und Kulturen						
	8.1 Baumschulen, Obstgärten, Rebberge					x
	8.2 Feldkulturen (Äcker)	x				
9 Bauten, Anlagen						
	9.1 Lagerplätze, Deponien				x	
	9.2 Bauten				x	
	9.3 Verkehrswege				x	
	9.4 Versiegeleter Sportplatz, Parkplatz etc.				x	

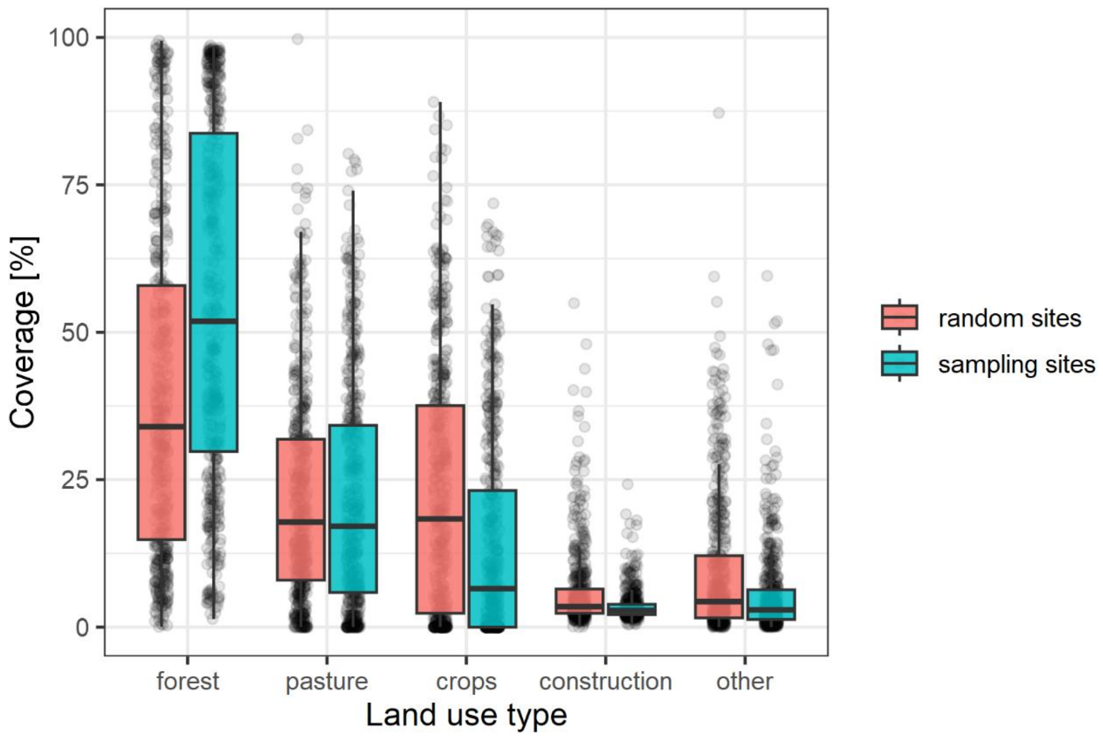

Fig. S1. Comparison of land use type contributions between sampling and random sites. Data for random sites was extracted from 484 arbitrarily set locations across the Swiss plateau. For both, random and sampling sites, data of percentage coverage per land use type was extracted from 600 m catchment buffers.

Table S2. Results for the spaMM spatial mixed-effect models (Gamma family with log-link) for relationships between land use type proportions and measured nitrate concentrations.

	**forest**				**pasture**				**crop**			
Predictors	*Est*	*S.E.*	*t*	*p-value*	*Est*	*S.E.*	*t*	*p-value*	*Est*	*S.E.*	*t*	*p-value*
(Intercept)	2.87	0.15	19.47	< 0.001	2.55	0.12	20.38	< 0.001	2.24	0.10	22.05	< 0.001
Land use proportion	- 0.0070	0.0025	- 2.78	0.0062	-0.00052	0.0037	- 0.14	0.89	0.015	0.0036	4.22	< 0.001
**Random Effects**												
ν, ρ	9.90, 0.0069				5.44, 0.0047				16.67, 0.010			
Random Effect Var. λ	0.55				0.59				0.50			
Residual Var. φ	0.023				0.023				0.021			
$N_{\text{Observations}}$	149				149				149			

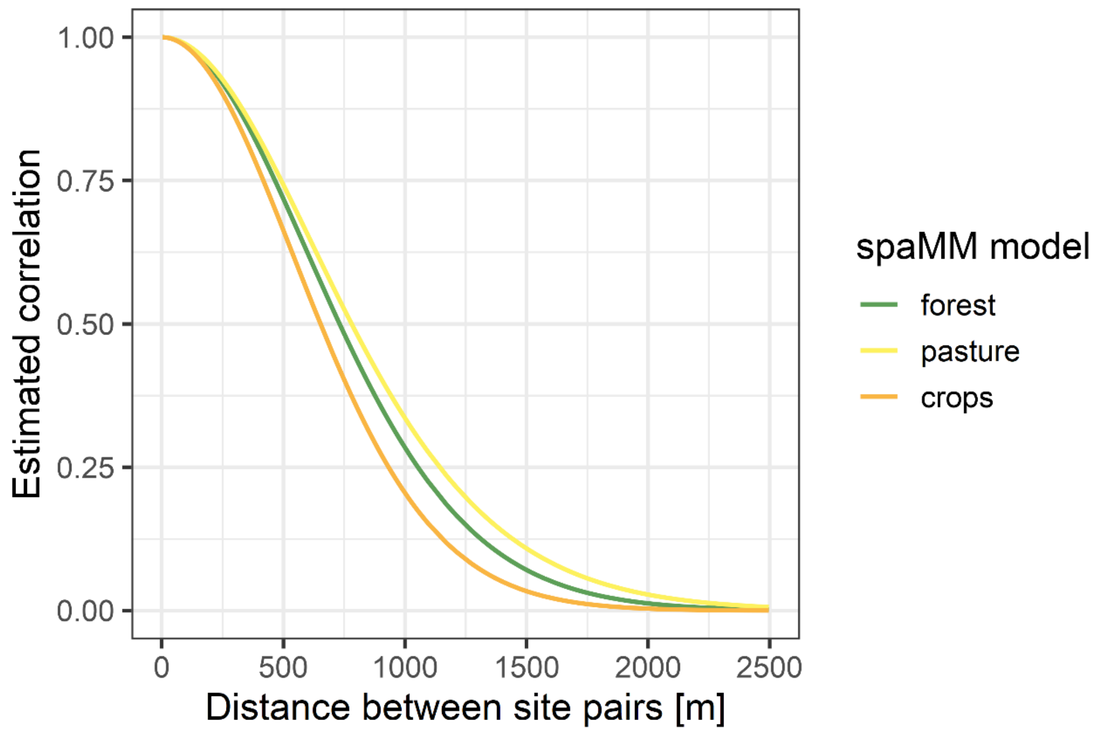

Fig. S2. Estimated spatial autocorrelation based on the spaMM spatial mixed-effect model results (Table S1) of relationships between land use type proportions and measured nitrate concentrations. Spatial autocorrelation was modeled including a spatially correlated random effect with Matérn covariance.

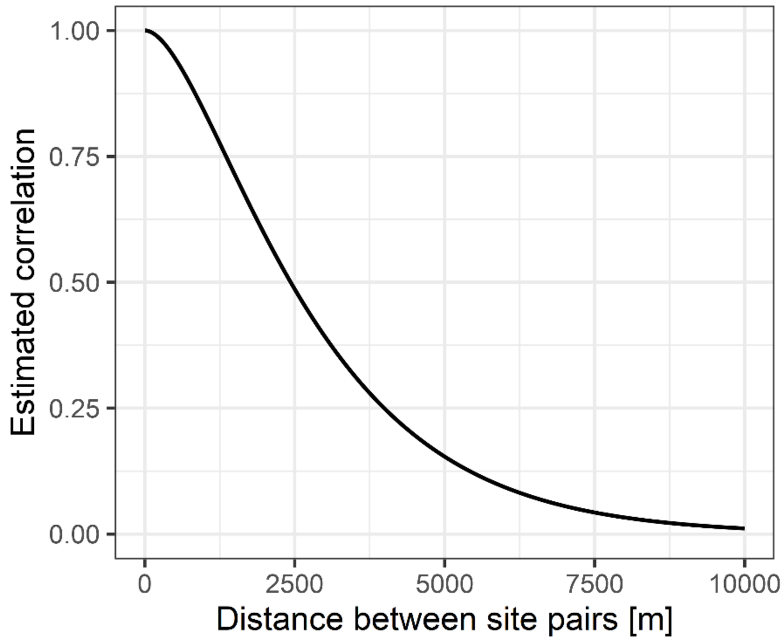

Fig. S3. Estimated spatial autocorrelation based on the spaMM spatial mixed-effect model results (Table 1) of the relationship between amphipod occurrence and environmental covariates including land use. Spatial autocorrelation was modeled including a spatially correlated random effect with Matérn covariance.

References

Price, B., Huber, N., Ginzler, C., Pazúr, R. & Rüetschi, M. (2021). The Habitat Map of Switzerland (v1). *EnviDat*, https://doi.org/10.16904/envidat.262.
